# Diagnostic Accuracy of the InBios Scrub Typhus Detect™ ELISA for the Detection of IgM Antibodies in Chittagong, Bangladesh

**DOI:** 10.3390/tropicalmed3030095

**Published:** 2018-09-01

**Authors:** Stuart D. Blacksell, Hugh W. F. Kingston, Ampai Tanganuchitcharnchai, Meghna Phanichkrivalkosil, Mosharraf Hossain, Amir Hossain, Aniruddha Ghose, Stije J. Leopold, Arjen M. Dondorp, Nicholas P. J. Day, Daniel H. Paris

**Affiliations:** 1 Mahidol-Oxford Tropical Medicine Research Unit, Faculty of Tropical Medicine, Mahidol University, 420/6 Rajvithee Road, Bangkok 10400, Thailand; hwfkingston@googlemail.com (H.W.F.K.); ampai@tropmedres.ac (A.T.); meghnakosil@hotmail.com (M.P.); Stije@tropmedres.ac (S.J.L.); arjen@tropmedres.ac (A.M.D.); nickd@tropmedres.ac (N.P.J.D.); daniel.paris@swisstph.ch (D.H.P.); 2 Centre for Tropical Medicine and Global Health, Nuffield Department of Clinical Medicine, University of Oxford, Old Road Campus, Roosevelt Drive, Oxford OX3 7FZ, UK; 3 Chittagong Medical College Hospital, Chittagong, Bangladesh; mosharraf144@gmail.com (M.H.); amir_hossain_ctg@yahoo.com (A.H.); anrdghs@yahoo.com (A.G.); 4 Department of Medicine, Swiss Tropical and Public Health Institute, 4056 Basel, Switzerland; 5 Faculty of Medicine, University of Basel, 4003 Basel, Switzerland

**Keywords:** scrub typhus, diagnosis, ELISA, Bangladesh

## Abstract

Here we estimated the accuracy of the InBios Scrub Typhus Detect™ immunoglobulin M (IgM) ELISA to determine the optimal optical density (OD) cut-off values for the diagnosis of scrub typhus. Patients with undifferentiated febrile illness from Chittagong, Bangladesh, provided samples for reference testing using (i) qPCR using the *Orientia* spp. 47-kDa *htra* gene, (ii) IFA ≥1:3200 on admission, (iii) immunofluorescence assay (IFA) ≥1:3200 on admission or 4-fold rise to ≥3200, and (iv) combination of PCR and IFA positivity. For sero-epidemiological purposes (ELISA vs. IFA ≥1:3200 on admission or 4-fold rise to ≥3200), the OD cut-off for admission samples was ≥1.25, resulting in a sensitivity (Sn) of 91.5 (95% confidence interval (95% CI: 96.8–82.5) and a specificity (Sp) of 92.4 (95% CI: 95.0–89.0), while for convalescent samples the OD cut-off was ≥1.50 with Sn of 66.0 (95% CI: 78.5–51.7) and Sp of 96.0 (95% CI: 98.3–92.3). Comparisons against comparator reference tests (ELISA vs. all tests including PCR) indicated the most appropriate cut-off OD to be within the range of 0.75–1.25. For admission samples, the best Sn/Sp compromise was at 1.25 OD (Sn 91.5%, Sp 92.4%) and for convalescent samples at 0.75 OD (Sn 69.8%, Sp 89.5%). A relatively high (stringent) diagnostic cut-off value provides increased diagnostic accuracy with high sensitivity and specificity in the majority of cases, while lowering the cut-off runs the risk of false positivity. This study underlines the need for regional assessment of new diagnostic tests according to the level of endemicity of the disease given the high levels of residual or cross-reacting antibodies in the general population.

## 1. Introduction

Scrub typhus, caused by *Orientia tsutsugamushi*, remains a significant acute febrile illness in most of Asia and northern Australasia, although recent studies indicate that its geographical range is expanding [1,2]. Without effective diagnostics, the delayed treatment of patients with febrile illnesses may result in complications leading to death [3]. The most accurate diagnosis relies on the combination of a method to detect the infectious agent such as a polymerase chain reaction (PCR) test, coupled with well-validated serological techniques [4]. Furthermore, the presence of clinical features increases diagnostic accuracy, that is, a thorough clinical examination to search for inoculation eschars will increase the likelihood of providing the correct diagnosis and treatment to the patient [5]. 

Diagnosis of scrub typhus is hampered by an absence of commercial and validated assays, leading to a tendency to use conventional technologies that are labor intensive and expensive, and lack standardization, such as the immunofluorescence assay (IFA) [6], due to issues related to diagnostic cut-off titers [7] and antigenic composition [8]. The in vitro culture of *O. tsutsugamushi* is not possible in the majority of locations, as it requires significant infrastructure, including continuous cell lines, experienced staff, and dedicated biosafety laboratories [9]. PCR is generally considered to be the most reliable diagnostic test upon admission; however, it requires significant levels of infrastructure and costly reagents, and despite improving the lower limits of detection, a positive result is only likely during the rickettsaemic phase of the infection [5]. Thus, combining PCR with antibody-based techniques may improve the possibility of detection. 

Antibody-based diagnostic assays play an important role in sero-prevalence studies to determine the epidemiology of scrub typhus, as well as in reference assays and point-of-care testing [10,11]. However, geographical locations of endemic disease patterns and their respective background cut-off levels for diagnosis require more consideration. This study evaluated the commercial InBios Scrub Typhus Detect™ immunoglobulin M (IgM) ELISA using a mixture of recombinant p56 kDa type-specific-antigens for the detection of *O. tsutsugamushi* IgM antibodies, to determine (a) its relationship to the current gold standard serological assay IFA, and (b) suitable diagnostic positivity cut-off levels for acute diagnostic and (c) for sero-epidemiology purposes based on a single serum admission sample at the scrub typhus-endemic locality of Chittagong, Bangladesh.

## 2. Materials and Methods

### 2.1. Samples

Patient recruitment and sample collection strategies have been described previously [12]. Briefly, patients admitted to the Chittagong Medical College Hospital (CMCH), Chittagong, Bangladesh, from August 2014 to September 2015 with an acute febrile illness and a history of fever for <3 weeks, and who were ≥12 years old, were enrolled in the study. Written informed consent was provided by all patients prior to their inclusion in the study, or by their relatives if the patient lacked the capacity to provide consent, or by their parents or guardians if their age was <16 years. Seventy-five serum samples were also collected from healthy controls. The study was approved by the CMCH ethics committee Bangladesh and the Oxford Tropical Research Ethics Committee (OxTREC) (study number: OxTREC 18-14). Admission and convalescent samples (7–14 days apart, where possible) were collected into ethylene diamine tetraacetic acid (EDTA) tubes and separated into packed cells and plasma prior to storage at −30 °C.

### 2.2. Reference Testing

Full details of the reference testing have been described previously [12]. Briefly, samples were tested with real-time PCR using the *Orientia* spp. 47-kDa *htra* gene and *Rickettsia* spp. 17-kDa gene. Positive results subsequently underwent confirmatory nested PCR assays with product sequencing, targeting the 56-kDa and 47-kDa gene targets for *Orientia* spp. and *ompB* qPCR, 17-kDa and *gltA* nPCR for *Rickettsia* spp., as previously described [13,14]. For serology, paired plasma samples were tested by scrub typhus indirect immunofluorescence assays using slides coated with *O. tsutsugamushi* (strains Karp, Kato, and Gilliam) as previously described [4,7,11]. A stringent diagnostic positivity criteria was either an admission IgM titer ≥1:3200 or a 4-fold rise to ≥1:3200 in the convalescent sample [11]. 

### 2.3. Scrub Typhus Detect™ IgM ELISA

The Scrub Typhus Detect™ IgM ELISA (Cat# STMS-1, InBios International Inc., Seattle WA, USA) uses recombinant p56kD type specific antigens of *Orientia tsutsugamushi* Karp, Kato, Gilliam, and TA716 strains to detect scrub typhus IgM antibodies. The manufacturer’s methods were followed exactly. All sera were tested at a 1:100 dilution and the results read at 450 nm using a microplate reader (Thermo Scientific™ Multiskan™ FC) to give a final optical density (OD) result (OD@450 nm). 

### 2.4. Analysis and Practical Assessment of Diagnostic Utility

To examine and compare the diagnostic utility of the InBios Scrub Typhus Detect™ IgM ELISA in a clinical setting, the following questions were posed and comparisons performed:

*Question 1. How do the InBios Scrub Typhus Detect™ ELISA results directly compare with the serological gold standard IFA?*

To determine the relationship between the InBios Scrub Typhus Detect™ IgM ELISA and the IgM IFA results, ELISA OD results and IFA endpoint titers for admission and convalescent samples were compared to determine a final Spearman correlation coefficient (*r*^2^).

*Question 2. For sero-epidemiology or screening purposes, what is the optimal diagnostic ELISA OD cut-off value compared to a range of IFA titers?*

To evaluate the suitability of the InBios Scrub Typhus Detect™ IgM ELISA for cross-sectional sero-prevalence studies, or for diagnostic screening purposes, we used two approaches; (1) application of the manufacturer’s suggested method using the mean OD of non-scrub typhus cases and the addition of 3 standard deviations (SD) to determine the positivity cut-off and, (2) IgM ELISA cut-offs with an optimal sensitivity (Sn) and specificity (Sp) were calculated by comparing a range of IFA cut-off values with the ELISA OD results for the admission samples.

*Question 3. In a patient presenting with suspected scrub typhus infection, how accurate are the ELISAs for the diagnosis of scrub typhus in absolute terms compared to reference assays (including non-serological assays) at various diagnostic cut-off values?*

The InBios Scrub Typhus Detect™ IgM ELISA results for all the admission samples were compared with all reference assay results including independent diagnostic tests to determine comparative diagnostic results. A range of ELISA cut-off values (0 OD to 3.0 OD) were compared against a composite of reference diagnostic modalities: (i) PCR alone, (ii) IFA ≥1:3200 on admission, (iii) IFA ≥1:3200 on admission or 4-fold rise to ≥3200, and (iv) combination of PCR and IFA positivity (i.e., i + ii + iii + iv are referred to as the modified scrub typhus infection criteria (mSTIC)) to determine Sn, Sp, and area under the receiver operator characteristic curve (AUROCC) values [11]. Analyses were performed using the *Diagt* and *ROCTAB* commands (Stata/IC 15.0 for Mac, Stata Corp., College Station, TX, USA).

## 3. Results

### 3.1. Reference Assay Results

#### 3.1.1. Patient Results

A total of 416 patients were enrolled into the study. The median number of days of fever prior to hospitalization was 10 (interquartile range: 7, 15). There were 412/416 (99%) of patient admission samples available for PCR or serology, and 253/412 (61%) patients were followed up to provide paired samples. Of the enrolled patients, 17% (71/412) had a diagnosis of scrub typhus by PCR and/or serology. Of the other rickettsial diseases tested, 3.6% patients (15/412) had *Rickettsia typhi* and 1.2% (5/412) *Rickettsia* spp. infections (Table 1).

#### 3.1.2. Characteristics of ELISA vs. IFA Results 

**Figure 1:**
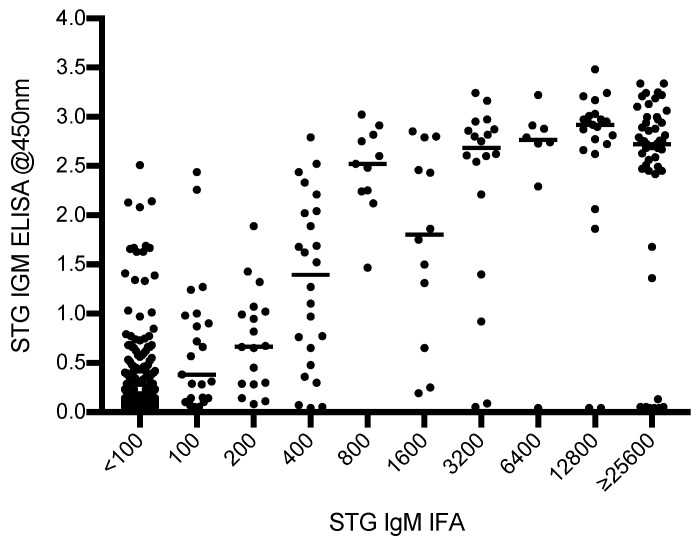
Distribution of InBios Scrub Typhus Detect™ immunoglobulin M (IgM) ELISA optical densities (ODs) compared with immunofluorescence assay (IFA) IgM for admission and convalescent samples.

Of the 665 samples tested (admission and convalescent), 476 samples gave IFA reciprocal titers of <1:100. The balance of the samples gave titers of 1:100 (*n* = 24), 1:200 (*n* = 18), 1:400 (*n* = 24), 1:800 (*n* = 11), 1:1600 (*n* = 12), 1:3200 (*n* = 18), 1:6400 (*n* = 8), 1:12,800 (*n* = 23), and ≥1:25,600 (*n* = 51). The distribution of the IFA titers with their relative ELISA ODs are presented in Figure 1.

*Question 1. Direct comparisons of ELISA with IFA results*

A limited relationship between ELISA OD and IFA IgM titers was demonstrated by a Spearman correlation coefficient *r*^2^ of 0.59; 7/420 (1.7%) samples had a very strongly positive IFA (≥25,600) but a very weak (<0.25) ELISA OD. 

*Question 2. For sero-epidemiology or screening purposes, what is the optimal diagnostic ELISA cut-off value compared to a range of IFA titers?*

Approach 1: 75 healthy controls were tested. Two outliers were removed from the analysis (ODs 2.69 and 1.94) because they were significantly beyond what would be considered normal values for healthy individuals. The mean and SD of the ODs were 0.259 and 0.251, respectively. Using the manufacturer’s cut-off formula of mean OD + 3 SD, the final ELISA OD cut-off value was 1.012, which was equivalent to an IgM IFA titre ≥3200. In the admission samples, Sn was 91.5 (95% confidence interval (95% CI): 96.8–82.5), Sp 90.9 (95% CI: 93.7–87.3), and AUROCC was 0.91 (95% CI: 0.95–0.88); for the convalescent samples, Sn was 66.0 (95% CI: 78.5–51.7), Sp 92.5 (95% CI: 95.7–87.9), and AUROCC was 0.79 (95% CI: 0.86–0.73), and for all samples Sn was 80.6 (95% CI: 87.2–72.6), Sp 91.5 (95% CI: 93.7–88.8), and AUROCC 0.86 (95% CI: (0.90–0.82).

**Figure 2:**
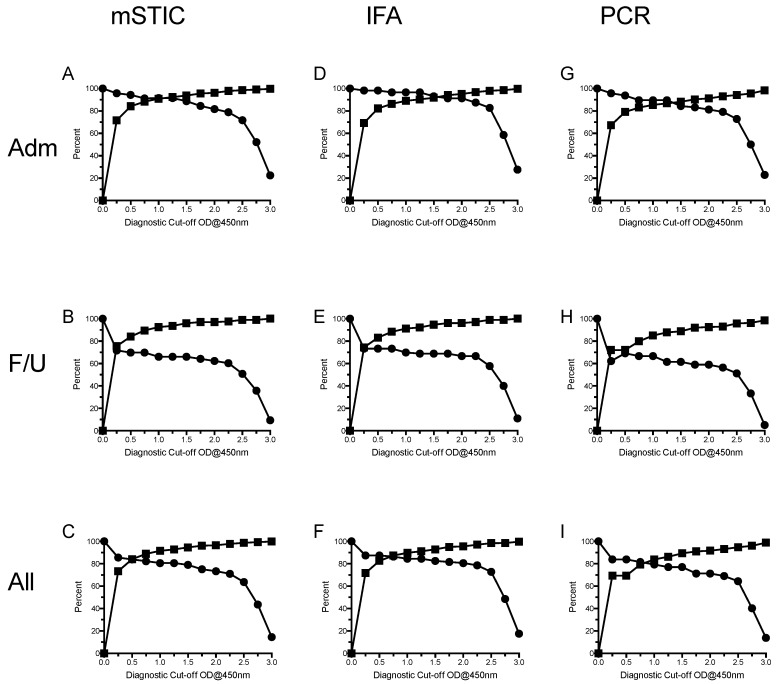
Sensitivity (●) and specificity (■) at a range of diagnostic cut-off values for the scrub typhus IgM ELISA when compared to the reference modified scrub typhus infection criteria (mSTIC), IFA, and PCR scrub typhus diagnosis for admission (Adm-**A**,**D**,**G**), convalescent (F/U-**B**,**E**,**H**), and all samples (**C**,**F**,**I**).

Approach 2: Using ELISA cut-off values (0 OD to 3.0 OD), diagnostic accuracy was assessed for an IgM IFA ≥1:3200 on admission or 4-fold rise to ≥3200 (Figure 2D–F). Considering all samples, Sn ranged from 14.5% (95% CI: 22.0–8.8) at ≥3.00 cut-off OD to 85.5% (95% CI: 91.2–78.0) at ≥0.25 cut-off OD; Sp ranged from 73.5% (95% CI: 77.1–69.4) at ≥0.25 cut-off OD to 99.9% (95% CI: 100–99.0) at ≥3.00 cut-off OD; AUROCC ranged from 0.57 (95% CI: 0.54–0.60) at ≥1.25 and 1.50 cut-off OD to 0.87 (95% CI: 0.90–0.83) at IFA ≥1:3200 on admission or 4-fold rise to ≥3200 cut-off OD. Generally, Sn demonstrated wider 95% CIs than did Sp, which is a function of the lower number of positive samples relative to negative samples. Similar results were generated for admission only and convalescent only samples (Figure 2D,E), although Sn for convalescent samples was lower due to a number of false negative results. 

For admission samples, the OD cut-off value providing highest Sn and Sp was ≥1.25, resulting in a Sn of 91.5 (96.8–82.5), Sp of 92.4 (95.0–89.0), and an AUROCC of 0.92 (0.96–0.88). For convalescent samples, this was ≥1.50 with a Sn of 66.0 (78.5–51.7), a Sp of 96.0 (98.3–92.3), and an AUROCC of 0.81 (0.88–0.74).

*Question 3. In a patient presenting with suspected scrub typhus infection, how accurate are the ELISAs for the diagnosis of scrub typhus in absolute terms when compared to reference assays at various diagnostic cut-off values?*

Examination of InBios Scrub Typhus Detect™ IgM ELISA results using mSTIC comparator reference results indicated that the selection of the most appropriate cut-off OD was in the range 0.75–1.25 (Table 2 and Figure 2A–C), with the exact cut-off value depending on a compromise between Sn and Sp (i.e., highest simultaneous Sn and Sp values). For admission samples, the best compromise Sn/Sp (Sn 91.5%, Sp 92.4%) was 1.25 OD (Figure 2A), with the same Sn but marginally higher Sp than at cut-off values 0.75 and 1.0 (90.9% and 88.3%, respectively) (Table 2). Considering the convalescent samples, the best compromise of Sn/Sp was 0.75 OD with values of 69.8% for Sn and 89.5% for Sp (Figure 2B). Considering all samples, the best compromise of Sn/Sp was 0.75 OD with values of 82.3% for Sn and 88.9% for Sp; however, increased cut-off values resulted in slightly lower Sn but increased Sp (Table 2 and Figure 2). Examination of subgroups of mSTIC data, IFA, and PCR demonstrated similar results (Figure 2D–I), and so did the reference mSTIC comparator with the exception that for admission samples, the IFA comparator results (Figure 2D) demonstrated higher sensitivities. 

## 4. Discussion

Here we estimated the accuracy of the InBios Scrub Typhus Detect™ IgM ELISA. The ELISA was compared with a number of diagnostic modalities to determine the optimal diagnostic cut-off values for acute scrub typhus diagnosis and cross-sectional sero-epidemiology studies using well-characterized, prospectively-collected samples from fever patients in Chittagong, Bangladesh. 

The diagnostic cut-off value suggested in this study (i.e., 0.75–1.25) is similar to that reported in previous research. Previous studies have examined the suitability of diagnostic cut-off values for the InBios Scrub Typhus Detect™ IgM ELISA in other geographic settings. The selection of the absolute cut-off value is dependent of the application (i.e., acute clinical diagnosis or cross-sectional sero-prevalence study) and the requirements for increased sensitivity or specificity, or a compromise between both. A study from Northern Thailand (Chiangrai) [15] determined that the OD cut-off value should be between 0.3 and 0.6 depending on the reference comparator and the cut-off threshold. Other studies conducted mainly in India using the same test have suggested cut-offs of 0.5 [16], 0.89 [17], and 1.0 [18]. The determination of the cut-off value is highly dependent on the samples assessed and the reference assays used. All of these studies highlighted the problems associated with high background antibody titers and the need to increase the cut-off to reduce false positivity. A few samples from patients with strongly positive IFA titres had negative InBios Scrub Typhus Detect™ IgM ELISA ODs. The reasons for this are unclear and may be related to antibodies reacting against targets other than the 56-kDa antigen, which is solely present in the ELISA. Results presented in this study demonstrate the difficulties associated with scrub typhus serology in an endemic setting. A recent evaluation of the most suitable positivity cut-off value for serological diagnosis in endemic settings demonstrated the need to increase the cut-off titer from the previously applied 1:400 [19] to reduce the likelihood of false-positive results. False-positive results may be due to a number of factors, including cross-reacting antibodies with related infectious agents such as other intracellular pathogens, non-specific immune stimulation, and residual or background antibodies from previous infections. Recent studies in Chiangrai, Thailand determined that diagnostic cut-off titers for scrub typhus IgM IFA needed to be increased to ≥1:3200 to reduce the possibility of false positivity to maintain acceptable levels of sensitivity and specificity [7]. 

In this study, we evaluated the results of the InBios Scrub Typhus Detect™ IgM ELISA against a group of conservative diagnostic modalities: (i) PCR alone, (ii) IFA ≥1:3200 on admission, (iii) IFA ≥1:3200 on admission or 4-fold rise to ≥3200, and (iv) combination of PCR and IFA positivity (i.e., i + ii + iii). Using a number of approaches including calculating the mean + 3 SD, comparing with a number of diagnostic modalities including reference serology, PCR, and a combination of both, it is clear that applying a relatively high (stringent) diagnostic cut-off value provides increased diagnostic accuracy with high sensitivity and specificity, in the majority of cases approaching 90% or greater. Lowering the diagnostic cut-off values and using those applied elsewhere runs the risk of false positivity and underlines the need for local assessment of new diagnostic tests where there is an endemic situation with high levels of residual antibodies in the general population.

The geographic variation in antigenic composition may contribute to discrepancies in assay specificity depending on local *O. tsutsugamushi* strains causing disease. The InBios Scrub Typhus Detect™ IgM ELISA purportedly uses a mixture of recombinant p56-kDa Karp, Kato, Gilliam, and TA716 strain antigens whilst the IFA slides used only have Karp, Kato, and Gilliam strains [8,15]. The *O. tsutsugamushi* strains that cause disease in Chittagong, Bangladesh have recently been genetically characterized as Karp, Gilliam, Kato, and TA763-like strains, with a dominance of Karp-like strains [12], although additional studies are required in other geographic centers. 

To gain a better understanding of the diagnostic characteristics of ELISA, novel diagnostic and other diagnostic modalities, additional prospective studies are required to validate proposed diagnostic cut-off values in various geographic settings.

## Figures and Tables

**Table 1:** Patient and diagnostic characteristics.

Diagnostic Test	Positive *n*	% Positive Overall
STG * polymerase chain reaction (PCR)	48	11.7%
STG immunoglobulin M (IgM) ≥3200 admission	54	13.1%
STG IgM 4-fold rise to ≥3200	8	1.9%
STG IgM ≥3200 or 4-fold rise to ≥3200	62	15.1%
Total STG positives (immunofluorescence assay (IFA) + PCR)	71	17.2%
TG ** PCR	15	3.6%
SFG *** PCR	5	1.2%

* STG–Scrub typhus group; ** STG–Typhus group; *** SFG–Spotted fever group.

**Table 2:** Sensitivity, specificity, and area under receiver operator characteristic curve (AUROCC) results for admission, convalescent, and all samples for a range of diagnostic cut-off values of the InBios Scrub Typhus Detect™ IgM ELISA for IFA ≥1:3200 on admission or 4-fold rise to ≥3200. The shaded sections indicate the suggested cut-off OD for Chittagong, Bangladesh.

Sample Timing	Cut-off OD	% Sensitivity (95% CI *)	% Specificity (95% CI)	AUROCC (95% CI)
Admission samples	≥0.00	100	0	
	≥0.25	95.8 (99.1–88.1)	71.6 (76.3–66.4)	0.84 (0.87–0.80)
	≥0.50	94.4 (98.4–86.2)	84.2 (87.9–79.8)	0.89 (0.93–0.86)
	≥0.75	91.5 (96.8–82.5)	88.3 (91.5–84.4)	0.90 (0.94–0.86)
	≥1.00	91.5 (96.8–82.5)	90.9 (93.7–87.3)	0.91 (0.95–0.88)
	≥1.25	91.5 (96.8–82.5)	92.4 (95.0–89.0)	0.92 (0.96–0.88)
	≥1.50	88.7 (95.0–79.0)	93.8 (96.1–90.7)	0.91 (0.95–0.87)
	≥1.75	84.5 (92.0–74.0)	95.6 (97.5–92.8)	0.90 (0.94–0.86)
	≥2.00	81.7 (89.9–70.9)	96.2 (98.0–93.6)	0.89 (0.94–0.84)
	≥2.25	78.9 (87.7–67.6)	97.9 (99.2–95.8)	0.88 (0.93–0.84)
	≥2.50	71.8 (81.9–59.9)	98.5 (99.5–96.6)	0.85 (0.91–0.80)
	≥2.75	52.1 (64.1–39.9)	99.1 (99.8–97.5)	0.76 (0.82–0.70)
	≥3.00	22.5 (34.0-13.5)	99.7 (100–98.4)	0.61 (0.66–0.56)
Convalescent samples	≥0.00	100	0	
	≥0.25	71.7 (83.2–57.7)	75.5 (81.3–68.9)	0.74 (0.80–0.67)
	≥0.50	69.8 (81.7–55.7)	84.0 (88.8–78.2)	0.77 (0.84–0.70)
	≥0.75	69.8 (81.7–55.7)	89.5 (93.4–84.4)	0.80 (0.86–0.73)
	≥1.00	66.0 (78.5–51.7)	92.5 (95.7–87.9)	0.79 (0.86–0.73)
	≥1.25	66.0 (78.5–51.7)	93.6 (96.5–89.1)	0.80 (0.86–0.73)
	≥1.50	66.0 (78.5–51.7)	96.0 (98.3–92.3)	0.81 (0.88–0.74)
	≥1.75	64.2 (76.9–49.8)	97.0 (98.9–93.6)	0.81 (0.87–0.74)
	≥2.00	62.3 (75.2–47.9)	97.0 (98.9–93.6)	0.80 (0.86–0.73)
	≥2.25	60.4 (73.5–46.0)	97.5 (99.2–94.3)	0.80 (0.86–0.73)
	≥2.50	50.9 (64.9–36.8)	99.0 (99.9–96.4)	0.75 (0.82–0.68)
	≥2.75	35.0 (50.2–23.1)	99.0 (99.9–96.4)	0.67 (0.74–0.61)
	≥3.00	9.4 (20.7–3.1)	100 (100–98.2)	0.55 (0.59–0.51)
All samples	≥0.00	100	0	
	≥0.25	85.5 (91.2–78.0)	73.4 (77.1–69.4)	0.79 (0.83–0.76)
	≥0.50	83.9 (89.9–76.2)	84.1 (87.1–80.7)	0.84 (0.88–0.80)
	≥0.75	82.3 (88.5–74.4)	88.9 (91.4–86.0)	0.86 (0.89–0.82)
	≥1.00	80.6 (87.2–72.6)	91.5 (93.7–88.8)	0.86 (0.90–0.82)
	≥1.25	80.6 (87.2–72.6)	92.8 (94.8–90.3)	0.87 (0.90–0.83)
	≥1.50	79.0 (85.8–70.8)	94.6 (96.4–92.4)	0.87 (0.91–0.83)
	≥1.75	75.0 (82.3–66.4)	96.1 (97.6–94.1)	0.86 (0.90–0.82)
	≥2.00	73.4 (80.9–64.7)	96.5 (97.9–94.6)	0.85 (0.89–0.81)
	≥2.25	71.0 (78.8–62.1)	97.8 (98.8–96.2)	0.84 (0.88–0.80)
	≥2.50	63.7 (72.2–54.6)	98.7 (99.5–97.4)	0.81 (0.86–0.77)
	≥2.75	43.5 (52.7–34.7)	99.3 (99.8–98.1)	0.71 (0.76–0.67)
	≥3.00	14.5 (22.0–8.8)	99.9 (100–99.0)	0.57 (0.54–0.60)

* 95% CI: 95% confidence interval.

## Abbreviations

qPCR	Quantitative polymerase chain reaction
95% CI	95% confidence interval
CMCH	Chittagong Medical College Hospital
STG	Scrub typhus group
TG	Typhus group
SFG	Spotted fever group
AUROCC	Area under receiver operator characteristic curve
mSTIC	Modified scrub typhus infection criteria
OxTREC	Oxford Tropical Research Ethics Committee
EDTA	Ethylene diamine tetraacetic acid

## References

[1] Weitzel T., Dittrich S., López J., Phuklia W., Martinez-Valdebenito C., Velásquez K., Blacksell S.D., Paris D.H., Abarca K. (2016). Endemic scrub typhus in South America. N. Engl. J. Med..

[2] Izzard L., Fuller A., Blacksell S.D., Paris D.H., Richards A.L., Aukkanit N., Nguyen C., Jiang J., Fenwick S., Day N.P.J. (2010). Isolation of a novel *Orientia* species (*O. chuto* sp. nov.) from a patient infected in Dubai. J. Clin. Microbiol..

[3] Taylor A.J., Paris D.H., Newton P.N. (2015). A systematic review of mortality from untreated scrub typhus (*Orientia tsutsugamushi*). PLoS Negl. Trop. Dis..

[4] Paris D.H., Blacksell S.D., Nawtaisong P., Jenjaroen K., Teeraratkul A., Chierakul W., Wuthiekanun V., Kantipong P., Day N.P.J. (2011). Diagnostic accuracy of a loop-mediated isothermal PCR assay for detection of *Orientia tsutsugamushi* during acute scrub typhus infection. PLoS Negl. Trop. Dis..

[5] Paris D.H., Dumler J.S. (2016). State of the art of diagnosis of rickettsial diseases: The use of blood specimens for diagnosis of scrub typhus, spotted fever group rickettsiosis, and murine typhus. Curr. Opin. Infect. Dis..

[6] Blacksell S.D., Bryant N.J., Paris D.H., Doust J.A., Sakoda Y., Day N.P.J. (2007). Scrub typhus serologic testing with the indirect immunofluorescence method as a diagnostic gold standard: A lack of consensus leads to a lot of confusion. Clin. Infect. Dis..

[7] Lim C., Paris D.H., Blacksell S.D., Laongnualpanich A., Kandipong P., Chierakul W., Wuthiekanun V., Day N.P.J., Cooper B.S., Limmathurotsakul D. (2015). How to determine the accuracy of an alternative diagnostic test when it is actually better than the reference tests: A re-evaluation of diagnostic tests for scrub typhus using Bayesian LCMs. PLoS ONE.

[8] James S.L., Blacksell S.D., Nawtaisong P., Nawtaisong A., Smith D.J., Day N.P.J., Paris D.H. (2016). Antigenic relationships among human pathogenic *Orientia tsutsugamushi* isolates from Thailand. PLoS Negl. Trop. Dis..

[9] Koh G.C., Maude R.J., Paris D.H., Newton P.N., Blacksell S.D. (2010). Diagnosis of scrub typhus. Am. J. Trop. Med. Hyg..

[10] Kingston H.W., Blacksell S.D., Tanganuchitcharnchai A., Laongnualpanich A., Basnyat B., Day N.P.J., Paris D.H. (2015). Comparative accuracy of the InBios Scrub Typhus Detect IgM rapid test for the detection of IgM antibodies by using conventional serology. Clin. Vaccine Immunol..

[11] Lim C., Blacksell S.D., Laongnualpanich A., Kantipong P., Day N.P.J., Paris D.H., Limmathurotsakul D. (2015). Optimal cutoff titers for indirect immunofluorescence assay for diagnosis of scrub typhus. J. Clin. Microbiol..

[12] Kingston H.W., Hossain M., Leopold S., Anantatat T., Tanganuchitcharncha A., Sinha I., Plewes K., Maude R.J., Hassan-Chowdhury M.A., Paul S. (2018). Rickettsial illnesses as important causes of febrile illness in Chittagong, Bangladesh. Emerg. Infect. Dis..

[13] Dittrich S., Phommasone K., Anantatat T., Panyanivong P., Slesak G., Blacksell S.D., Dubot-Pérès A., Castonguay-Vanier J., Stenos J., Newton P.N. (2014). *Rickettsia felis* infections and comorbid conditions, Laos, 2003–2011. Emerg. Infect. Dis..

[14] Mayxay M., Castonguay-Vanier J., Chansamouth V., Dubot-Pérès A., Paris D.H., Phetsouvanh R., Tangkhabuanbutra J., Douangdala P., Inthalath S., Souvannasing P. (2013). Causes of non-malarial fever in Laos: A prospective study. Lancet Glob. Health.

[15] Blacksell S.D., Tanganuchitcharncha A., Nawtaisong P., Kantipong P., Laongnualpanich A., Day N.P.J., Paris D.H. (2015). Diagnostic accuracy of the InBios Scrub Typhus Detect enzyme-linked immunoassay for the detection of IgM antibodies in Northern Thailand. Clin. Vaccine Immunol..

[16] Morch K., Manoharan A., Chandy S., Chacko N., Alvarez-Uria G., Patil S., Henry A., Nesaraj J., Kuriakose C., Singh A. (2017). Acute undifferentiated fever in India: A multicentre study of aetiology and diagnostic accuracy. BMC Infect. Dis..

[17] Gupta N., Chaudhry R., Thakur C.K. (2016). Determination of cutoff of ELISA and immunofluorescence assay for scrub typhus. J. Glob. Infect. Dis..

[18] Koraluru M., Bairy I., Varma M., Vidyasagar S. (2015). Diagnostic validation of selected serological tests for detecting scrub typhus. Microbiol. Immunol..

[19] Coleman R.E., Sangkasuwan V., Suwanabun N., Eamsila C., Mungviriya S., Devine P., Richards A.L., Rowland D., Ching W.M., Sattabongkot J. (2002). Comparative evaluation of selected diagnostic assays for the detection of IgG and IgM antibody to *Orientia tsutsugamushi* in Thailand. Am. J. Trop. Med. Hyg..

